# Unraveling the Molecular Mechanisms of Fructus Anisi Stellati as a Remedy for Infantile Colic by Network Pharmacology

**DOI:** 10.1155/2020/9210304

**Published:** 2020-12-17

**Authors:** Xingyu Li, Yan Xu

**Affiliations:** ^1^Department of Chemistry, Cleveland State University, Cleveland, OH 44115, USA; ^2^College of Science, Yunnan Agricultural University, Kunming 650201, China

## Abstract

Fructus anisi stellati (FAS) is an anise-scented star-shaped fruit from *Illicium verum* tree. It is commonly consumed in many cultures as food and medicine, particularly as a remedy for infantile colic (IC). The elucidation of molecular mechanisms of action would contribute to the understanding of the traditional therapy of FAS and help to guide the preclinical and clinical study of this herb. The aim is to investigate the key therapeutic compounds of FAS and to explore the underlying molecular mechanisms of FAS therapy. The chemical compounds of FAS were obtained through data mining on TCMSP and ADME screening, and the common targets of the FAS compounds and the IC-correlated diseases were obtained from PharmMapper, GeneCards, and OMIM databases. GO and KEGG databases were used for molecular function and pathway enrichment. Cytoscape was used for network construction and analysis. SystemsDock was used for molecular docking. Three key compounds (*i.e*., quercetin, luteolin, and kaempferol), 19 targets, 7 molecular pathways, and 12 IC-correlated diseases were identified to be involved in the molecular mechanisms of FAS for the treatment of IC. This work showed that three therapeutic modules were primarily engaged in the molecular mechanisms of FAS for IC therapy, including the inhibition of inflammatory reactions, stimulating immunoglobulin A (IgA) production in the gastrointestinal tract, and enhancing the secretion of digestive enzymes.

## 1. Introduction

Infantile colic (IC) describes a symptom complex of excessive and inconsolable crying in babies that is a common phenomenon in infancy. Although IC is usually a self-limiting condition, it is a source of extreme distress for the infant, parents, family, and health-care professionals [1]. At present, the specific focus or nature of IC remains unclear. Various etiologic factors have been proposed to contribute to this disorder, including environmental, psychosocial, physical, neurodevelopmental, nutritional, and gastrointestinal factors. Although no firm conclusions can be drawn paving the way for a multifactorial explanation for this entity, as the academic purposes the Rome IV criteria define IC as a functional gastrointestinal disorder from birth to five months of age [1, 2]. Several possible gastrointestinal factors have been suggested to contribute to the pathophysiology of IC including cow's milk protein intolerance, gastroesophageal reflux, excessive intestinal gas, lactose intolerance, and gut hormones [1]. Herbal medicine is a popular strategy applied for the management of IC caused by gastrointestinal factors [3, 4], which contain multiple components such as fennel [5].

Fructus anisi stellati (FAS) is an anise-scented star-shaped fruit of *Illicium verum* Hook. f. belonging to the Schisandraceae family [6], according to the plant list (http://www.theplantlist.org), commonly known as star anise or Chinese star anise, and is an aromatic medium-sized evergreen tree, native to northeast Vietnam and southwest China [7], and distributed in North America, Atlantic region, and tropical and subtropical zones of Asia [8]. FAS is commonly known as being safe and nontoxic when consumed as both food and medicine in many cultures [9]. It has traditionally been used as a flavor ingredient in daily cuisine recognized in China as far back as 100 B.C. It has also been used in medicine throughout Asia and North America for many illnesses [10], in which it has been widely used to treat childhood colic, abdominal pain, colitis, diarrhea, and bloating [11]. While FAS is commonly used, and its impact is appealing as an important traditional Chinese medicine (TCM), the potential molecular mechanisms of its effect on IC were not evident. Multicomponent and multitarget are the significant features of TCM, which have made the molecular mechanism analysis complex and challenging. Fortunately, network pharmacology has been proven to be a suitable method to explore the underlying mechanism between TCM and known targets systematically. Based on the interaction among active components of TCM and their protein targets, as well as relevant biological functions and pathways, a network pharmacology study enables us to investigate the possible molecular mechanisms of a TCM to a particular disease [12].

In the current studies, the targets of FAS active compounds and IC-correlated pathways were carefully evaluated based on the network pharmacology to systematically explore the prospective targets and molecular mechanism and provide a hypothesis for IC therapeutic research and clinical study.

## 2. Materials and Methods

### 2.1. Screening Candidate Compounds in FAS

Forty-nine compounds of FAS (Table S1) were obtained from the Traditional Chinese Medicine Systems Pharmacology (TCMSP) database (http://lsp.nwu.edu.cn/tcmsp.php) which is a distinctive Chinese herbal medicines pharmacology platform that captures drug, target, and disease interactions [13]. Then, the candidate compounds were screened using two ADME (short for Absorption, Distribution, Metabolism, and Excretion) models, including oral bioavailability (OB) and drug-likeness (DL). The threshold values for these screening models are set to OB ≥ 30% and DL ≥ 0.18, respectively [14].

### 2.2. Prediction Targets of Candidate Compounds

The targets of FAS compounds were retrieved from the TCMSP and searched in the UniProt (http://www.uniprot.org) database for human-correlated protein codes. Together, an online target prediction platform PharmMapper (http://lilab-ecust.cn/pharmmapper/) was also used to retrieve the targets of FAS compounds with a “fit score”  > 4 [15].

### 2.3. Target Genes of IC-Correlated Diseases

The target genes of IC-correlated diseases were gathered from the GeneCards database (https://www.genecards.org/) [16] and the OMIM database (http://www.omim.org/) [17]. The search keywords were “colitis OR diarrhea OR “lactose intolerance” OR “infantile colic” OR “abdominal pain” OR “inflammatory bowel disease (IBD)” with the relevance score >5.

### 2.4. Common Targets of the FAS Compounds and IC-Correlated Diseases

The common targets of the FAS compounds and IC-correlated disease were identified by Venn analysis (http://bioinfogp.cnb.csic.es/tools/venny/). These common targets were further cross-referenced with David (https://david-d.ncifcrf.gov/) and PDB (http://www.rcsb.org/pdb/gene/) [18]. Cytoscape software (https://cytoscape.org/,version.3.8.0) was used to construct and visualize the interaction network of FAS compounds and the targets of IC.

### 2.5. Protein-Protein Interaction Data

The common targets were used to construct the protein-protein interaction (PPI) network using the String database (https://string-db.org/) with setting up multiple proteins and *Homo sapiens*, and confidence scores >0.7.

### 2.6. GO and KEGG Enrichment Analyses

To systematically elucidate the molecular interaction network of common targets, Gene Ontology (GO) molecular function and Kyoto Encyclopedia of Genes and Genomes (KEGG) pathway enrichment analyses were performed using *R* (version 3.5.2) software packages with *p* < 0.05 (http://www.bioconductor.org/) [19]. The significant molecular functions and pathways of the target genes were extracted.

### 2.7. Networks Construction and Analyses

To visualize and analyze various relationships among FAS, active compounds, targets, pathways, and diseases, Cytoscape was used to construct and analyze networks, including compounds-targets (C-T) interaction network and compounds-targets-pathways-diseases (C-T-P-D) interaction network. The Cytoscape plugin Network Analyzer was used for network topological analysis.

### 2.8. Molecular Docking Simulation

Molecular docking was performed to confirm the binding properties of active compounds and key IC-correlated targets using SystemsDock [20].

## 3. Results and Discussion

### 3.1. The Workflow

**Figure 1:**
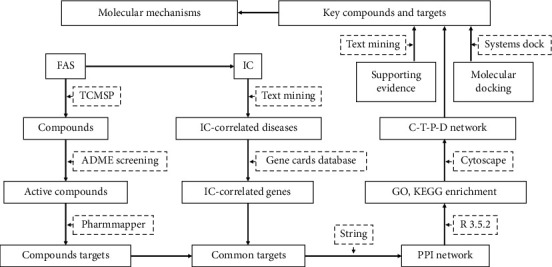
The workflow of the network pharmacology study of FAS.

A schematic diagram of the present study is shown in Figure 1. Firstly, the common targets were identified between the predicted targets of the active compounds and the IC-correlated genes. Then, the common targets were subjected to enrichment analyses for protein molecular functions and molecular pathways. Furthermore, the network between compounds, targets, pathways, and diseases was constructed to analyze the underlying molecular mechanism. Finally, molecular docking was performed to evaluate the binding properties between active compounds and targets, and supporting evidence to the proposed molecular mechanisms was provided.

### 3.2. Candidate Compounds in FAS

**Figure 2:**
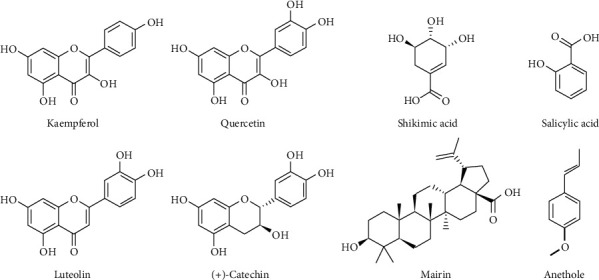
The chemical structures of eight selected bioactive compounds of FAS.

There were eight candidate compounds, including five (mairin, luteolin kaempferol, (+)-catechin, and quercetin) which passed the ADME screening criteria and three (anethole, salicylic acid, and shikimic acid) selected based on the reported biological activities. For example, anethole, a type of aromatic compound that generally exists in nature as one of the essential oils, had a significant relaxing effect on tracheal and ileal smooth muscles, anti-inflammatory property, and rapid absorbability when orally administered [21]. Their chemical structures and ADME parameters were shown in Figure 2 and Table 1, respectively.

### 3.3. Common Targets of Candidate Compounds and IC

Based on the 8 candidate compounds, a total of 250 putative targets were retrieved from PharmMapper and TCMSP after removing duplicates (Table S2). There were 1,074 IC-correlated genes obtained from the GeneCard database and OMIM after removing duplicates (Table S3). Between the putative targets of the candidate compounds and IC-correlated genes, 92 common targets were identified (Table S4).

### 3.4. PPI Network of Common Targets

**Figure 3:**
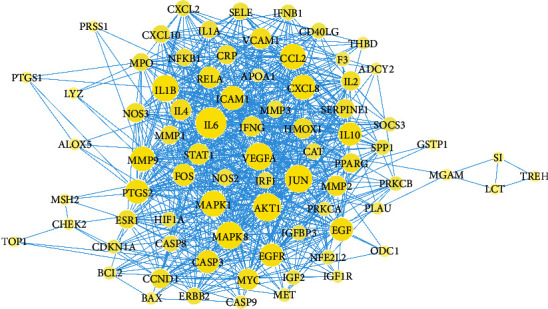
The PPI network of the common targets of the selected bioactive compounds and IC-correlated diseases. The larger the yellow nodes and the darker the blue edges, the higher the centrality of the nodes in the network.

**Figure 4:**
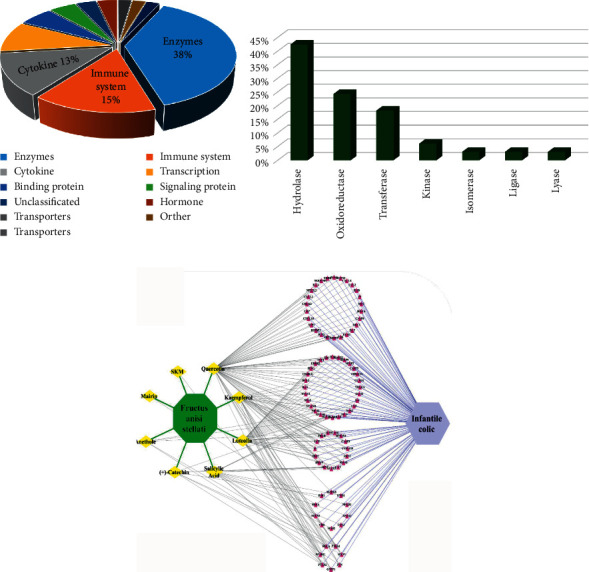
Compound-target (C-T) network analysis. (a) Distribution of biochemical categories among the common targets. (b) Classification of targets in the enzyme group. (c) C-T network, where FAS is colored green, the compounds are colored yellow, the targets are colored red, and the disease is colored purple.

The common targets were used to construct the PPI network using String. Among the 92 common targets, 86 (Table S5) were associated with each other with a minimum of two connections and a confidence score >0.7, which were considered as significant targets of IC. As shown in Figure 3, the PPI network had 86 nodes and 779 edges. The larger the nodes are or the more the edges are, the higher the degree of centrality the nodes have and the more important the nodes are in the network. The biochemical classifications of these 86 significant targets include enzymes, immune system, cytokine, and transcription factors (Figure 4(a)). Among them, 38.4% of 86 targets are enzymes including 14 hydrolases, 8 oxidoreductases, 6 transferases, 2 kinases, 1 isomerase, 1 ligase, and 1 lyase (Figure 4(b)). These enzymes play critical functions in biological processes. For example, gastric and pancreatic lipases play the main role in gastrointestinal digestion of nutritional fat [11], and about 40% of infants with IC suffered from a lactase deficiency [22].

### 3.5. Compound-Target (C-T) Network Analysis

The eight candidate compounds and 86 significant targets were used to construct a C-T network using Cytoscape (Figure 4(c)). The network consists of 96 nodes (86 targets, 8 compounds, 1 plant, and 1 disease) and 260 edges, of which 166 edges are formed between the compound and the target. According to the ranking of centrality degree, the top 3 ranked compounds were quercetin, luteolin, and kaempferol with degree of centrality values of 69, 39, and 29, respectively. These 3 compounds bound to 76 important targets accounted for 88.4% of the total targets. These 3 compounds belong to a class of plant secondary metabolites known as flavonoids with various pharmacological activities [23]. Therefore, quercetin, luteolin, and kaempferol were considered as the key active compounds, and the 76 targets were considered as targets for further KEGG and GO enrichment analyses.

### 3.6. Enrichment Analyses and Therapeutic Modules

GO enrichment analysis was performed on 76 targets and 102 GO molecular functions were obtained (*p* < 0.05) (Table S6). The 102 GO molecular functions were found to belong to 6 functional categories including 53 binding activities, 26 catalytic activities, 12 molecular function regulators, 8 transcription regulator activities, 2 antioxidant activities, and 1 molecular transducer activity.

KEGG enrichment analysis was also performed on 76 targets and resulted in 113 molecular pathways (*p* < 0.05) (Table S7). By searching the KEGG database, 288 diseases were found (Table S8). Among these findings, 7 molecular pathways (Table 2) and 12 diseases were correlated with IC (Table 3), which led to three therapeutic modules for the treatment of IC-correlated diseases.

Module I consisted of four pathways (i.e., hsa04973, hsa00052, hsa00500, and hsa04972) related to carbohydrate and fat digestion, absorption, and metabolism. For examples, hsa04973 is associated with congenital glucose-galactose malabsorption (H01261); and hsa00052 is related to galactosemia (H00070), congenital lactase deficiency (H00116), and galactose-1P uridylyltransferase deficiency (H02008), whereas hsa00500 is connected to congenital sucrase-isomaltase deficiency (H00115) and trehalase deficiency (H02090); and hsa04972 is related to Type 2 diabetes mellitus (H00409) and pancreatic lipase deficiency (H02330). Module II consisted of two pathways (hsa05321 and hsa04672) related to Crohn disease (H00286), IBD (H01227), and ulcerative colitis (H01466). Module III consisted of one pathway (hsa04060) related to cytokine-cytokine receptor interaction and associated with Crohn disease (H00286), Type 1 diabetes mellitus (H00408), IBD (H01227), and ulcerative colitis (H01466).

### 3.7. C-T-P-D Network Analysis

**Figure 5:**
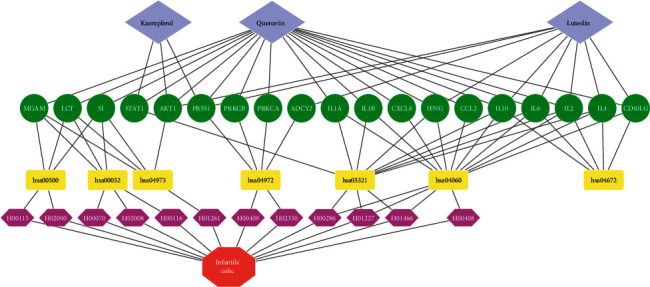
The compound-target-pathway-disease (C-T-P-D) network. In the first row, the gray diamonds represent the compounds, the second-row green ellipses represent the targets, the third-row yellow rectangles represent the biological pathways, the fourth-row pink hexagons represent the diseases, and the fifth-row red octagon represents the infantile colic.

The C-T-P-D network (Figure 5) was constructed using Cytoscape with 3 key compounds, 12 IC-correlated diseases, 7 relevant molecular pathways, and 19 targets associated with the molecular pathways. As shown in Figure 5, each pathway in the network regulates one or more IC-correlated diseases. Some protein targets (*i.e*., SI, LCT, MGAM, IL6, IL4, IL2, and IL10) are shared by multiple pathways. Furthermore, the three key compounds, quercetin, luteolin, and kaempferol, bind to 18 (94.7%), 9 (47.4%), and 3 (15.8%) out of the 19 targets suggesting the key therapeutic role of quercetin, and the supporting roles of luteolin and kaempferol in treating IC.

### 3.8. Molecular Docking

The bindings of quercetin, luteolin, and kaempferol to 19 targets were further verified by molecular docking with SystemsDock. The binding strength of a target-compound complex was evaluated with a docking score. As shown in Table 4, all 48 combinations of target-compound pairs showed docking scores more than a cutoff value at 5.52, indicating strong binding between a compound ligand and a protein target [20]. These results further confirmed that quercetin, luteolin, and kaempferol are the key ligand to the targets with good binding properties.

### 3.9. Supporting Evidence for FAS Key Compounds as Therapeutic Agents

Quercetin was reported to possess antiulcer, antioxidant, antidiabetic, and anti-inflammatory properties [24, 25] and could modulate some key regulatory enzymes in humans such as alkaline phosphatase and lens aldose reductase [26]. Besides, it was reported that quercetin can increase the expression of lactase [27] and inhibit the release of proinflammatory mediators and the expression of inflammatory proteins like adhesion molecules, cyclooxygenase, and nitric oxide synthase [28]. Recent research shows that quercetin can increase the secretion of immunoglobulin *A* (IgA) [29]. Quercetin could affect the progression of colitis and IBD [30] and possessed protective and beneficial effects on chronic intestinal inflammation [31]. It was used to treat inflammatory illnesses caused by mast cells [32, 33], treat IBD induced by *Citrobacter rodentium* [34], and effectively decrease oxidative stress and inflammatory damage to both ileum and colon tissues [35]. Luteolin was reported to have strong anti-inflammatory activity [36, 37] and strong radical scavenging and cell-protective properties [38]. It was considered as a therapeutic agent for IBD [39], and inflammation-related diseases in humans [40]. Kaempferol also showed anti-inflammatory activities and immunomodulatory effects [41].

## 4. Conclusion

In this work, we have investigated the molecular mechanisms of FAS for the treatment of IC with a network pharmacology approach. The active compounds of FAS were selected through the ADME screening of the FAS compounds from the TCMSP database. The common targets of the active compounds and IC-correlated diseases were obtained from PharmMapper and GeneCards and used to construct the PPI network. Through GO and KEGG enrichment analyses, the seven molecular pathways that were associated with three key compounds, 19 targets, and 12 IC-correlated diseases were extracted and used to construct the C-T-P-D network. The network analysis revealed that FAS compound quercetin is the key therapeutic agent, whereas luteolin and kaempferol are the regulating and modulating agents in treating IC. The therapeutic effect of FAS on IC was based on the synergistic effect of multiple compounds acting on multiple targets through various therapeutic modules including the inhibition of inflammatory reactions, stimulating IgA production in the gastrointestinal tract, and enhancing the secretion of digestive enzymes. Our findings were supported by the molecular docking analysis, and the experimental results from the literature search. This work provides a mechanistic guide for preclinical and clinical studies of FAS on IC therapy.

## Figures and Tables

**Table 1:** Selected bioactive compounds of FAS and their ADME parameters.

No.	Compounds	OB (%)^a^	DL^b^	HL (h)^c^
1	Mairin	55.38	0.78	8.87
2	Luteolin	36.16	0.25	15.94
3	Kaempferol	41.88	0.24	14.74
4	(+)-Catechin	54.83	0.24	0.61
5	Quercetin	46.43	0.28	14.40
6	Anethole	32.49	0.03	1.68
7	Salicylic acid	32.13	0.03	12.00
8	(−)-Shikimic acid	46.24	0.04	11.18

^a^OB, oral bioavailability, ^b^DL, drug-likeness, and ^c^HL, half-life.

**Table 2:** The KEGG enrichment analysis for IC-correlated pathways.

Pathway ID	Description	Gene ID
hsa04060	Cytokine-cytokine receptor interaction	IL4, IFNB1, TNFSF15, IL1B, IL10, IL6, IL2, IFNG, CD40LG, CCL2, CXCL8, IL1A, CXCL11, CXCL2, CXCL10
hsa05321	Inflammatory bowel disease (IBD)	RELA, IL4, NFKB1, IL1B, IL10, JUN, IL6, IL2, IFNG, STAT1, IL1A
hsa04972	Pancreatic secretion	CHRM3, CTRB1, PRSS3, PRSS1, ADCY2, PRKCA, PRKCB
hsa04672	Intestinal immune network for IgA production	IL4, IL10, IL6, IL2, CD40LG
hsa04973	Carbohydrate digestion and absorption	AKT1, PRKCB, MGAM, HK2
hsa00052	Galactose metabolism	MGAM, LCT, SI
hsa00500	Starch and sucrose metabolism	MGAM, SI, TREH

**Table 3:** The IC-correlated diseases from KEGG database.

Disease ID	Description
H00070	Galactosemia
H00115	Congenital sucrase-isomaltase deficiency; disaccharide intolerance I
H00116	Congenital lactase deficiency; disaccharide intolerance II
H00286	Crohn disease
H00408	Type 1 diabetes mellitus
H00409	Type 2 diabetes mellitus
H01227	Inflammatory bowel disease (IBD)
H01261	Congenital glucose-galactose malabsorption
H01466	Ulcerative colitis
H02008	Galactose-1P uridylyltransferase deficiency; classic galactosemia
H02090	Trehalase deficiency
H02330	Pancreatic lipase deficiency

**Table 4:** The docking scores obtained by SystemsDock for the binding interactions between target proteins and key compounds.

No.	Protein name	PDB ID^a^	Test compounds	Docking scores (pK_d_)^b^
1	STAT1	1BF5	Luteolin	7.897
2	STAT1	1BF5	Kaempferol	7.875
3	STAT1	1BF5	Quercetin	7.810
4	IL1A	5UC6	Luteolin	7.696
5	IL1A	5UC6	Quercetin	7.576
6	IL1A	5UC6	Kaempferol	7.523
7	IL10	2ILK	Luteolin	7.311
8	ADCY2	1AB8	Kaempferol	7.057
9	ADCY2	1AB8	Luteolin	6.989
10	ADCY2	1AB8	Quercetin	6.982
11	IL10	2ILK	Kaempferol	6.779
12	IL10	2ILK	Quercetin	6.721
13	CCL2	1DOK	Kaempferol	6.690
14	IL6	1ALU	Quercetin	6.684
15	IFNG	6E3K	Quercetin	6.680
16	IFNG	6E3K	Kaempferol	6.678
17	IL2	5UTZ	Kaempferol	6.677
18	SI	3LPO	Kaempferol	6.672
19	IFNG	6E3K	Luteolin	6.670
20	IL1B	5MVZ	Quercetin	6.656
21	CD40LG	3LKJ	Luteolin	6.655
22	IL2	5UTZ	Quercetin	6.655
23	IL2	5UTZ	Luteolin	6.652
24	IL1B	5MVZ	Kaempferol	6.647
25	IL6	1ALU	Kaempferol	6.644
26	CCL2	1DOK	Quercetin	6.624
27	AKT1	3OCB	Quercetin	6.622
28	IL6	1ALU	Luteolin	6.622
29	CD40LG	3LKJ	Quercetin	6.610
30	IL1B	5MVZ	Luteolin	6.608
31	PRKCB	2I0E	Luteolin	6.607
32	CCL2	1DOK	Luteolin	6.599
33	AKT1	3OCB	Luteolin	6.530
34	CD40LG	3LKJ	Kaempferol	6.511
35	PRKCA	4RA4	Kaempferol	6.452
36	PRKCA	4RA4	Luteolin	6.442
37	AKT1	3OCB	Kaempferol	6.436
38	PRKCA	4RA4	Quercetin	6.416
39	SI	3LPO	Quercetin	6.415
40	SI	3LPO	Luteolin	6.397
41	PRKCB	2I0E	Quercetin	6.359
42	IL4	5FHX	Quercetin	6.301
43	IL4	5FHX	Luteolin	6.265
44	PRKCB	2I0E	Kaempferol	6.256
45	IL4	5FHX	Kaempferol	6.156
46	PRSS1	4WWY	Luteolin	6.044
47	PRSS1	4WWY	Kaempferol	5.939
48	PRSS1	4WWY	Quercetin	5.850

^a^PDB ID, protein data bank identifier. ^b^pK_d_, the negative logarithm of dissociation constant.

## Data Availability

The data used to support the findings of this study are available from the corresponding author upon request.

## Supplementary Materials

Supplementary Materials Table S1: the ADME parameters of the identified components of FAS and their chemical structures; Table S2: the predicted binding proteins and gene symbols of eight selected bioactive components of FAS; Table S3a: the genes of IC-associated diseases obtained from GenCards database; Table S3b: the genes of IC-associated diseases obtained from OMIM database; Table S4: key information of the common genes obtained by intersecting the genes of component binding proteins and IC-associated diseases; Table S5: the topological features of all nodes in the IC targets' PPI network constructed by string website; Table S6: the GO molecular function enrichment analysis for selected targets; Table S7. the KEGG pathway enrichment analysis for selected targets; Table S8: the KEGG pathways and all related diseases.
